# Risks and Resources for Depressive Symptoms and Anxiety in Children and Adolescents During the COVID-19 Pandemic – Results of the Longitudinal COPSY Study

**DOI:** 10.3389/fpsyt.2022.901783

**Published:** 2022-07-07

**Authors:** Neslihan Güzelsoy, Ulrike Ravens-Sieberer, Joachim Westenhöfer, Janine Devine, Michael Erhart, Heike Hölling, Anne Kaman

**Affiliations:** ^1^Department of Child and Adolescent Psychiatry, Psychotherapy, and Psychosomatics, University Medical Center Hamburg-Eppendorf, Hamburg, Germany; ^2^Department of Health Sciences, Faculty of Life Sciences, Competence Center Health, Hamburg University of Applied Sciences, Hamburg, Germany; ^3^Department of Public Health, Alice Salomon University of Applied Science, Berlin, Germany; ^4^Department of Psychology, Apollon University of Applied Science, Bremen, Germany; ^5^Department of Epidemiology and Health Monitoring, Robert Koch Institute, Berlin, Germany

**Keywords:** COVID-19, mental health, risk factors, resource factors, children and adolescents

## Abstract

**Background:**

Mental health during the COVID-19 pandemic is of particularly high relevance. Especially for children and adolescents, the pandemic and its restrictions represent a significant burden. The present study aims to identify risks and resources for depressive symptoms and anxiety in children and adolescents during the pandemic in Germany.

**Materials and Methods:**

Self-reported data from the first wave of the longitudinal COVID-19 and Psychological Health (COPSY) study were used to investigate risks and resources among *n* = 811 children and adolescents aged 11–17 years. Depressive symptoms and anxiety were measured at the first follow-up 6 months later. Multivariate linear regression analyses were performed to investigate the effects of risks and resources on depressive symptoms and anxiety.

**Results:**

Parental depressive symptoms predicted depressive symptoms and anxiety in children and adolescents 6 months later. Female gender was identified as a risk factor for anxiety during the pandemic. None of the potential resources were associated with depressive symptoms or anxiety at the follow-up.

**Conclusion:**

The findings provide evidence of risk factors for depressive symptoms and anxiety during the COVID-19 pandemic. Children and adolescents who face risk factors need to be identified early and monitored during the pandemic. Family-based intervention programs are needed to help vulnerable children and adolescents cope with the challenges of the pandemic.

## Introduction

The challenges and restrictions resulting from the COVID-19 pandemic can have a negative impact on mental health (1, 2). Studies from throughout the world have shown the adverse effects of the COVID-19 pandemic on the mental health of children and adolescents (3–6). Findings from the population-based longitudinal COVID-19 and Psychological Health (COPSY) study show that a majority of children and adolescents in Germany have felt burdened by the pandemic (1). The lack of contact with friends and homeschooling due to school closures represents an additional burden (7, 8). Family life was negatively affected as conflicts within the family increased and escalated more often (8, 9). The COPSY study found that during the pandemic health-related quality of life decreased and the stress level, sleeping problems, loneliness, hyperactivity/inattention, conduct problems, and psychosomatic symptoms increased (8, 10, 11). Of particular note is the significant increase in symptoms of depression and anxiety in children, which seem associated with pandemic measures (12–16).

Mental health problems in children may persist into adolescence and pose risk factors for the emergence of mental disorders in adulthood (17–19). Therefore, information on risk and resource factors that influence the development of mental health problems such as depressive and anxiety symptoms during the COVID-19 pandemic is crucial.

Existing research identifies risks and resources that are associated with the occurrence of mental problems such as depression and anxiety in children and adolescents. In terms of social risk and resources, studies indicate that social isolation and loneliness as well as lack of peer relationships are linked to depression and anxiety during the COVID-19 pandemic (2, 14, 20). Moreover, excessive or insufficient demands at school and educational failure have been associated with an increased risk of depression (21). School and kindergarten ideally are a resource and can provide social support, foster emotional development and coping with trauma (22, 23). Traumas like domestic violence, abuse or neglect, which increased during the COVID-19 pandemic (2, 24), can lead to adverse long-term effects such as mental problems in adulthood (25, 26). Especially for traumatized children, social and emotional support can protect against depressive symptoms (27). Studies show that peers, school and good relationships between children and teachers have a promoting effect on mental resilience (28–30).

Regarding familial risks, parental stress and psychopathology play a key role in children’s mental health (31, 32). Conflicts with parents and a negative family climate have an adverse impact on emotional health (33, 34). Children and adolescents whose mothers suffer from depression and those who have a negative mother-child relationship are at higher risk of developing internalizing problems such as depression (35–37). Conversely, a two-parent family structure, family cohesion and positive parenting are well-established resource factors for ameliorating mental problems (28, 36, 38).

Among personal factors, overall worse psychological health and preexisting psychiatric disorders present important risk factors for the deterioration of mental health (39). Quittkat et al. showed that the COVID-19 pandemic exacerbated the degree of symptom severity in children and adolescents with a history of mental problems (40). Self-esteem, a positive self-concept and an optimistic attitude (34, 38) as well as successful coping mechanisms (41, 42) can be considered resources for the mental health of youth.

Besides the psychosocial risks and resources, there are biological and sociodemographic factors associated with depressive symptoms and anxiety in youth. Female gender and puberty increase the risk of internalizing symptoms (43). Further, a low socioeconomic status as well as a migration background are strongly associated with psychological problems (8, 28, 44, 45). A study undertaken in Brazil during the COVID-19 pandemic demonstrated that anxiety rates are higher among children with parents who have essential jobs and who are separated from their parents (46). Quarantined youth living in high epidemic areas (47), who have a high-risk family member (48) or relatives infected with COVID-19 (49) also experienced higher rates of anxiety (6, 50, 51).

Overall, previous research has examined factors influencing the development of depressive symptoms and anxiety in youth before and at the beginning of the COVID-19 pandemic. However, there is a lack of knowledge regarding risks and resources for children and adolescents over the course of the pandemic based on longitudinal studies.

Therefore, the present population-based longitudinal study aims to determine which familial, personal as well as social risk and resources at the early stage of the pandemic predict depressive symptoms and anxiety over the course of the pandemic, i.e., 6 months later. Our hypotheses were that conflicts within the family, school stress and parental depressive symptoms (risk factors) predict depressive symptoms and anxiety in children and adolescents during the pandemic. Further, we assumed that a positive family climate, personal resources and social support (resource factors) are associated with fewer depressive symptoms and anxiety.

## Materials and Methods

### Study

The population-based longitudinal COPSY study examines the mental health and quality of life of children and adolescents aged 7–17 years during the COVID-19 pandemic in Germany. The design and methodology of the COPSY study is conceptualized based on the population-based longitudinal BELLA study. The BELLA study presents the mental health module of the German National Health Interview and Examination Survey for Children and Adolescents (KiGGS) (52). The first wave of the COPSY study was conducted from May 26th to June 10th, 2020. At that time Germany was under a partial nationwide lockdown, with educational institutions, leisure and cultural facilities partly closed or restricted. In total, *n* = 1,586 parents of children aged 7–17 years as well as *n* = 1,040 children and adolescents aged 11–17 years took part in the online survey. The second wave of the COPSY study took place from December 17th, 2020 to January 25th, 2021, during the second wave of the pandemic. At that time, there was a complete nationwide lockdown, with schools as well as leisure and cultural facilities closed. Families who had participated in the first wave of the COPSY study were re-contacted, informed about the second wave and asked for their consent. Overall, *n* = 1,288 families participated in the second wave (re-participation rate of 85.1%). A responder versus non-responder analysis revealed no significant differences in sociodemographic or health-related variables. The samples match the sociodemographic characteristics (age, gender, education, region) of the German population according to the Microcensus of 2018. The COPSY study was approved by the Local Psychological Ethics Committee and the Commissioner for Data Protection of the University of Hamburg. Detailed information on the design, methods and results of the first two waves of the COPSY study can be found elsewhere (1, 53).

### Participants

In the present analysis, data from the baseline survey (wave 1) and the first follow-up (wave 2) of the COPSY study were used. Participants were included in the analysis if (i) they were 11–17 years old at baseline, provided self-reports, and (ii) their data of depressive and anxiety symptoms were available at follow-up. This resulted in a final sample of *n* = 811 children and adolescents.

### Measures

#### Sociodemographic Variables

The self-report version of the online survey included questions on age and gender of the children and adolescents. In addition, the parent-reported version included further sociodemographic information on the educational and migration background of the parents. Socio-demographic data collected at baseline were defined as control variables in the regression analysis. Parental education was assessed in accordance with the Comparative Analyses of Social Mobility in Industrial Nations (CASMIN) classification. For this purpose, the highest academic and vocational qualifications of both parents were assessed by two items (54). A categorization into three status groups (low, medium and high parental education) was performed for sample description and further analysis. In addition, information on migration background was gathered by asking the parents two questions regarding their migration status.

#### Risk Factors

Conflicts within the family were assessed by one newly developed item (“To what degree did the frequency of arguments in your family change compared to before the pandemic?”) with a five-point response scale (1 = *a lot more* to 5 = *much less).* The variable was dichotomized into 0 = family conflicts did not increase (summarizing the response options *much less*, *a little less* and *just as much*) and 1 = family conflicts increased (combining the response options *a lot more* and *a little more*). School burden was also measured by one newly developed item (“How do you perceive schooling and learning/work now compared to a regular school or workday?”) presented with a five-point response scale (1 = *a lot more difficult* to 5 = *much less difficult*). The variable was dichotomized into 0 = school was not perceived as more stressful (combining the response options *much less difficult*, *a little less difficult* and *both equal*) and 1 = school was perceived as more stressful (summarizing the response options *a lot more difficult* and *a little more difficult*). Parental depressive symptoms were assessed by the eight-item Patient Health Questionnaire (PHQ-8), which is a valid diagnostic and severity measure for depressive disorders used in population-based studies (55). Each item (e.g., “Over the last week, how often have you been bothered by any of the following problems? Feeling down, depressed or hopeless”) was offered with four response options ranging from 1 = *not at all* to 4 = *nearly every day*). A scale sum score was calculated with values ranging from 0 to 24. A higher score indicates more severe depressive symptoms of parents.

#### Resource Factors

Personal resources were measured using five self-reported items administered in the KiGGS study (56). The scale captures individual capabilities such as self-efficacy, optimism, and a positive self-concept. The items (e.g., “I look to the future with optimism/confidence”) were provided with four-point response options (1 = *not true* to 4 = *exactly true*). The whole scale comprises total scores ranging from 5 to 20, with higher scores reflecting more pronounced personal resources. Four self-report items from the Family Climate Scale (FSC) were administered to assess family cohesion (57). The FSC collects data on sense of belonging and cohesiveness of individual family members. Each item (e.g., “In our family everybody cares about each other’s worries”) was provided with four-point response options (1 = *not true* to 4 = *exactly true*). The scale sum score ranges from 4 to 16. A higher sum score reflects a stronger family cohesion. Data on social support were collected using four self-report items from the German translation of the Social Support Scale (SSS) (58). The items (e.g., “How often has there been someone you can count on to listen to you when you need to talk”) were answered by children and adolescents using a five-point response options (1 = *never* to 5 = *always*). The scale comprises total values from 4 to 20, with higher scores demonstrating more social support.

#### Depressive Symptoms

The German version of the Center for Epidemiological Studies Depression Scale (CES-DC) was administered to assess self-reported depressive symptoms in children and adolescents at baseline and at follow-up 6 months later (59). The scale consists of seven items (e.g., “I felt sad”) offered with a four-point response scale (1 = *rarely or not at all* to 4 = *mostly, all the time*). A scale sum score was calculated with values ranging from 7 to 28. A higher score indicates more depressive symptoms.

#### Anxiety

To measure anxiety in children and adolescents at baseline and at follow-up 6 months later, the subscale for generalized anxiety from the Screen for Child Anxiety Related Disorders (SCARED) questionnaire was applied as self-report version (60). It includes nine items (e.g., “I worry about what is going to happen in the future”) presented with three response options (0 = *not true or hardly ever true* to 2 = *very true or often true*). The sum scale score ranges from 0 to 18, with higher scores indicating more anxiety.

### Data Analysis

Descriptive analyses were carried out covering the calculation of frequencies, means and standard deviations of all variables. Bivariate analyses (chi-square tests, Spearman and Pearson correlations) were performed to examine associations between the predictor variables. Furthermore, linear regression analyses were conducted to determine to what degree the predictors can be considered as risk or resource factors for anxiety and depressive symptoms in children and adolescents. First, the association between each of the predictors and anxiety and depressive symptoms was examined using univariate linear regression analyses. Second, two multivariate linear regression models were carried out (one for anxiety and one for depressive symptoms). All predictor and control variables such as age, gender, parental education, and migration background were entered simultaneously into the regression models. For all regression analyses, metric variables were centered around the mean. All statistical analyses were performed using the SPSS version 25.

## Results

### Sample Characteristics

The analyzed sample including *n* = 811 children and adolescents aged 11–17 years (50.3% female) is described in Table 1. The majority of the children and adolescents were German (86.3%). The parents had a medium educational level (53.1%). The mean score for depressive symptoms at baseline was *M* = 11.35 and for anxiety *M* = 5.74. Among the risk factors, school stress was reported most often at 64.2%. Slightly more than a quarter of the children and adolescents reported an increase in family conflicts (26.4%). The mean scores of parental depressive symptoms was *M* = 5.17. Among the resource factors, the mean scores for personal resources were *M* = 15.30, for family climate *M* = 12.92 and for social support *M* = 16.35. Six months later, the mean score for depressive symptoms among the children and adolescents was *M* = 11.85 and *M* = 6.08 for anxiety.

**TABLE 1:** Description of the analyzed sample of children and adolescents.

	Total (*n* = 811)	
	*n (%)*	*M* (SD)
**Control variables**		
** Gender**		
Female	408 (50.3%)	
Male	403 (49.7%)	
Age (in years)		14.27 (1.87)
** Parental education**		
Low	164 (20.2%)	
Medium	431 (53.1%)	
High	203 (25.0%)	
** Migration background**		
Yes	111 (13.7%)	
No	700 (86.3%)	
Depressive symptoms at baseline		11.35 (3.68)
Anxiety at baseline		5.74 (4.38)
**Risk factors**		
Family conflicts	214 (26.4%)	
School burden	521 (64.2%)	
Parental depressive symptoms	119 (14.7%)	5.17 (4.85)
**Resource factors**		
Personal resources		15.30 (2.73)
Family climate		12.92 (2.32)
Social support		16.35 (2.82)
**Outcome variables (6 months later)**		
Depressive symptoms		11.85 (4.05)
Anxiety		6.08 (4.42)

*n, number of participants; M, mean; SD, standard deviation; Missing values were given for n = 13 for parental education.*

### Correlations Between Predictors

The correlations between the control and predictor variables are shown in Table 2. The sociodemographic variables showed only weak associations with one another and with the risk and resource factors, with correlation coefficients less than *r* = 0.20. Depressive symptoms at baseline were strongly correlated with anxiety at baseline (*r* = 0.600, *p* < *0.001*). Both depressive symptoms at baseline (*r* = –0.552, *p* < *0.001*) as well as anxiety at baseline (*r* = –0.458, *p* < *0.001*) showed strong correlations with personal resources. Among the risk factors, the highest intercorrelation was found between family conflicts and family climate (*r* = –0.291, *p* < *0.001*) and between parental depressive symptoms and children’s depressive symptoms at baseline (*r* = 0.399, *p* < *0.001*). In terms of resource factors, the strongest positive relationships were detected between family climate and personal resources (*r* = 0.486, *p* < *0.001*) and between family climate and social support (*r* = 0.567, *p* < *0.001*).

**TABLE 2:** Zero-order correlations among predictor variables.

Variables	1.	2.	3.	4.	5.	6.	7.	8.	9.	10.	11.	12.
1. Gender^a^	–											
2. Age^c^	0.00	–										
3. Parental education^b^	−0.72*	0.08*	–									
4. Migration background^a^	1.96	–0.04	0.11**	–								
5. Depressive symptoms at baseline^c^	0.09*	–0.02	0.08*	0.04	–							
6. Anxiety at baseline^c^	0.16***	–0.05	0.03	–0.05	0.60***	–						
7. Family conflicts^a^	1.49	−0.08*	0.06	0.40	0.23***	0.15***	–					
8. School burden^a^	0.10	−0.08*	0.52	0.13	0.24***	0.12***	25.75***	–				
9. Parental depressive symptoms^c^	0.01	–0.03	−0.09*	0.02	0.40***	0.26***	0.23***	0.18***	–			
10. Personal resources^c^	−0.10**	0.09**	0.02	–0.01	−0.55***	−0.46***	−0.21***	0.20***	−0.25***	–		
11. Family climate^c^	0.02	–0.02	−0.16***	−0.09**	−0.41***	−0.28***	−0.29***	–0.06	−0.19***	0.49***	–	
12. Social support^c^	–0.01	−0.08*	−0.14***	−0.07*	−0.41***	−0.25***	−0.20***	–0.06	−0.17***	0.35***	0.56***	–

*Zero-order correlations are presented by pearson correlations for continuous variables, point-biserial correlations for one dichotomous and one continuous variable, spearman correlations for one ordinal and continuous variable and chi-square tests for dichotomous variables.*

*^a^Dichotomous variable; ^b^Ordinal variable; ^c^Continuous variable.*

*p ≤ 0.05; **p ≤ 0.01; ***p ≤ 0.001.

### Univariate Linear Regression Analyses

The results of the univariate regression analyses are presented in Supplementary Tables 1 and 2. The findings indicate that among the sociodemographic variables, only female gender was significantly associated with depressive symptoms (ß = 0.11, *p* = *0.002*) and anxiety (ß = 0.16, *p* < *0.001*) 6 months later. Depressive symptoms at baseline (ß = 0.60, *p* < *0.001*) and anxiety at baseline (ß = 0.45, *p* < *0.001*) were positively associated with depressive symptoms at follow-up. In addition, depressive symptoms at baseline (ß = 0.48, *p* < *0.001*) and anxiety at baseline (ß = 0.64, *p* < *0.001*) also showed positive associations with anxiety at follow-up. An examination of the risk factors revealed that all the risk factors proved to be significant predictors of later depressive symptoms and anxiety. Parental depressive symptoms were most strongly associated with depressive symptoms (ß = 0.39, *p* ≤ 0.001) and anxiety (ß = 0.27, *p* ≤ 0.001) at follow-up. In terms of resource factors, all were associated with fewer depressive symptoms and anxiety 6 months later. Personal resources demonstrated the strongest negative association with depressive symptoms (ß = –0.41, *p* ≤ 0.001) and anxiety (ß = –0.39, *p* ≤ 0.001) at follow-up.

### Multivariate Linear Regression Analyses

The results of the multivariate linear regression analyses are presented in Tables 3 and 4. Among the sociodemographic variables, female gender proved to be a significant risk factor for later anxiety (ß = 0.07, *p* = *0.012*). Findings from both models indicated that parental depressive symptoms were significantly positively associated with depressive symptoms (ß = 0.18, *p* < *0.001*) and anxiety (ß = 0.08, *p* = *0.011*) among youth 6 months later, which is in line with results of the univariate analysis. In addition, depressive symptoms at baseline (ß = 0.38, *p* < *0.001*) as well as anxiety as baseline (ß = 0.13, *p* < *0.001*) significantly predicted later depressive symptoms. Anxiety at baseline (ß = 0.53, *p* < *0.001*) was strongly associated with anxiety at follow-up. Among the resource factors, none were significantly associated with depressive symptoms or anxiety 6 months later.

**TABLE 3:** Results of the multivariate linear regression analyses; risk and resource factors in children and adolescents predicting depressive symptoms 6 months later.

	*b*	ß	*p*
*Constant*	11.834		<0.001
**Control variables**			
Gender (female)	0.392	0.048	0.082
Age	0.099	0.045	0.102
**Parental education (reference: high)**			
Low	–0.228	–0.023	0.497
Medium	–0.118	–0.015	0.655
Migration background	0.243	0.021	0.455
Depressive symptoms at baseline	0.414	0.376	<0.001
Anxiety at baseline	0.116	0.125	<0.001
**Risk factors**			
Family conflicts	0.270	0.029	0.318
School burden	–0.277	–0.033	0.252
Parental depressive symptoms	0.148	0.178	<0.001
**Resource factors**			
Personal resources	–0.092	–0.062	0.083
Family climate	–0.040	–0.023	0.524
Social support	–0.091	–0.064	0.062
Model fit	*F*[13] = 43.06		
	*p* < 0.001		
	*adjusted R*^2^ = *0.403*		

*Outcome: depressive symptoms; n = 811.*

**TABLE 4:** Results of the multivariate linear regression analyses; risk and resource factors in children and adolescents predicting anxiety 6 months later.

	*b*	ß	*p*
*Constant*	5.915		<0.001
**Control variables**			
Gender (female)	0.602	0.068	0.012
Age	0.069	0.029	0.284
** Parental education (reference: high)**			
Low	–0.108	–0.010	0.762
Medium	–0.182	–0.021	0.516
Migration background	0.436	0.034	0.207
Depressive symptoms at baseline	0.088	0.073	0.061
Anxiety at baseline	0.540	0.534	<0.001
**Risk factors**			
Family conflicts	0.031	0.003	0.931
School burden	–0.135	–0.015	0.599
Parental depressive symptoms	0.068	0.075	0.011
**Resource factors**			
Personal resources	–0.089	–0.055	0.114
Family climate	–0.098	–0.051	0.146
Social support	–0.012	–0.007	0.823
Model fit	*F*[13] = 49.38		
	*p* < 0.001		
	*adjusted R*^2^ = *0.437*		

*Outcome: anxiety; n = 811.*

## Discussion

The aim of this study was to identify risk and resource factors for depressive symptoms and anxiety in children and adolescents during the COVID-19 pandemic. Findings of the population-based longitudinal COPSY study revealed that parental depressive symptoms were associated with stronger depressive symptoms and anxiety in children and adolescents during the pandemic. Other than expected none of the potential resources were associated with depressive symptoms or anxiety during the pandemic in our multivariate model.

Findings of the univariate regression analyses showed that the examined risk factors such as family conflicts, school burden and parental depressive symptoms were positively associated with stronger depressive symptoms and anxiety in children and adolescents during the pandemic. However, most of the identified risk factors did not predict later depressive symptoms and anxiety in our multivariate analysis. This might be due to the fact that in the interaction of several risk factors, family conflicts and school burden lose their significance. Further, controlling for depression and anxiety scores at baseline means that we modelled the change in depression and anxiety scores from baseline to follow-up. Thus, it is likely that predictors that are important for the occurrence of depression or anxiety *per se* might not turn out to be significant regarding the change in these outcomes.

In the multivariate model, only parental depressive symptoms emerged as a risk factor for depressive symptoms and anxiety in children and adolescents 6 months later. This finding is in line with previous studies investigating the relationship between parental psychopathology and mental disorders in children and adolescents (32, 36, 37).

In the univariate regression analyses, the examined factors such as personal resources, family climate and social support were significantly associated with fewer depressive symptoms and anxiety 6 months later. However, we found no direct effects of those resource factors in our multivariate model. Nevertheless, the important role of family cohesion in the context of internalizing mental health problems has been well examined in previous studies (35, 61, 62). Our results of the univariate regression analyses thus underline the importance of family-based intervention programs during the COVID-19 pandemic to support a positive family climate and strengthen family cohesion to mitigate the adverse effects of potential risk factors. Furthermore, special mental health counseling and support services should be offered for parents with depressive symptoms or mental health issues as well as for children and adolescents with pre-existing psychological health problems to cope with the additional burden of the pandemic.

Regarding the examined control variables, we found that depressive symptoms and anxiety at baseline were associated with depressive symptoms at follow-up. Moreover, anxiety at baseline predicted anxiety at follow-up. This finding is in line with the fact that mental disorders are highly recurrent and persistent and often last into adulthood (63). In addition, female gender was significantly associated with stronger symptoms of anxiety at follow-up, which is consistent with previous studies on gender distribution in internalizing disorders indicating that females are about twice as likely to experience anxiety compared to males (64, 65).

The present study shows the following limitations. The variables included in our multivariate regression analyses explained 40.3% of the variance in depressive symptoms and 43.7% of the variance in anxiety. Although this is a sizeable proportion, these findings may suggest that the development of depressive symptoms and anxiety during the COVID-19 pandemic is related to other important factors that we did not take into account in our models. These factors can be genetic risks, environmental aspects such as living conditions or the experience of stressful life events, domestic violence, and abuse (66). These aspects should be investigated in future studies. Moreover, it should be noted that the population-based COPSY study cannot identify causal relationships, as it is an observational study that only detects associations between risk and resource factors. Furthermore, it cannot be ruled out that other events occurred between the baseline and follow-up that influenced the development of depressive symptoms and anxiety during the pandemic. With regard to the assessment of depressive symptoms and anxiety measured by established and validated screening questionnaires it should be noted that these instruments are mostly not suitable for clinical diagnoses of mental health problems. The results of the COPSY study are neither generalizable nor transferable to other countries due to possible differences in study design and methodology as well as existing differences in conditions during the pandemic such as infection rates, lockdown measures and access to health care services.

The present study has the following strengths. First of all, the COPSY study is one of the first population-based longitudinal studies on health-related quality of life and mental health in children and adolescents with two measurement points during the COVID-19 pandemic. The study provides important findings on psychosocial risks and resources for the development of depressive symptoms and anxiety in children and adolescents during the pandemic, that are highly relevant for prevention, clinical practice and policy. The strengths of the COPSY study include the wide age range of the participants and the longitudinal analysis over a pandemic time period of 6 months. We examined a large population-based sample of *n* = 811 children and adolescents including their parents. Established self- and proxy-report measures were used to assess a range of psychosocial risk and resource factors. The self-reported data allowed insight into the perspective of children and young people.

Overall, the findings of the present study revealed that parental depressive symptoms were associated with stronger depressive symptoms and anxiety in children and adolescents during the COVID-19 pandemic. Given the high number of adults affected by depression and anxiety during the pandemic, the overall poorer mental health of parents is of particular concern for the emotional health of children and young people. Compared to pre-pandemic data showing a prevalence of 10.1% for depression among adults in Germany (67), the prevalence in this study, also assessed with the PHQ-8, is slightly higher at 14.7% during the pandemic. Other studies have also shown that parents reported higher rates of depression and anxiety during the pandemic compared to pre-pandemic estimates (68, 69). Mothers seem to be particularly impacted, most likely due to a higher burden caused by household chores, homecare/schooling and home office during lockdown phases (70, 71). These results are in line with several studies from Germany (72–74) as well as international reviews (75–78) reporting high prevalence rates for depression and anxiety in the general population during the pandemic.

Further, girls seem to be at higher risk than boys for developing anxiety during the pandemic. Special focus should also be placed on children and adolescents who have already shown depressive symptoms and anxiety at the start of the pandemic. These children and adolescents at higher risk for developing mental health problems during the COVID-19 pandemic need to be detected at an early stage to avoid mental health problems exacerbate into clinical mental disorders. Based on our findings, we recommend the implementation of low-threshold prevention and intervention measures such as resource-oriented and family-based as well as parent support programs in communities, kindergartens and schools to target this high risk youth. In addition, in the planning of lockdown measures such as school closures or social distancing, the mental health of children and adolescents should be carefully taken into account. Particularly politicians as well as education and healthcare professionals should be aware of the importance of preventive measures for child and adolescent mental health during the COVID-19 pandemic. The latter need to be empowered by financial resources to plan and implement such measures. Future research is needed to identify further risk and resource factors for the development of mental health problems in children and adolescents over the course of the pandemic, so that prevention and intervention measures can be adapted to the specific burden and needs of children and adolescents to help them cope with these challenging times.

## Data Availability Statement

The raw data supporting the conclusions of this article will be made available by the authors, without undue reservation.

## Ethics Statement

The COPSY study was approved by the Local Psychological Ethics Committee and the Commissioner for Data Protection of the University of Hamburg, Germany. Written informed consent to participate in this study was provided by the participants’ legal guardian/next of kin.

## Author Contributions

NG performed the statistical analyses, interpreted the data, and wrote the first draft of the manuscript. UR-S and AK were principle investigators of the COPSY study, responsible for its design, funding, general decisions of measurement, supervised data cleaning and preparation, and revised the manuscript critically. JW, JD, ME, and HH revised the manuscript critically. All authors contributed to the article and approved the final manuscript.

## Conflict of Interest

The authors declare that the research was conducted in the absence of any commercial or financial relationships that could be construed as a potential conflict of interest.

## Publisher’s Note

All claims expressed in this article are solely those of the authors and do not necessarily represent those of their affiliated organizations, or those of the publisher, the editors and the reviewers. Any product that may be evaluated in this article, or claim that may be made by its manufacturer, is not guaranteed or endorsed by the publisher.
