# Supplementary material for: Risks and Resources for Depressive Symptoms and Anxiety in Children and Adolescents During the COVID-19 Pandemic – Results of the Longitudinal COPSY Study
Source: Front Psychiatry. 2022 Jul 7;13:901783. doi: 10.3389/fpsyt.2022.901783 (PMC9301280; doi:10.3389/fpsyt.2022.901783)
Supplement: Supplementary file 1 [file Table_1.DOCX]

Supplementary Material

Supplementary Table 1. Results of the univariate linear regression analyses; Risk and resource factors in children and adolescents predicting depressive symptoms six months later

|  |  | | |
| --- | --- | --- | --- |
|  | ***b*** | **ß** | ***p*** |
| **Control variables** |  |  |  |
| Gender (female) | .860 | .106 | .002 |
| Age | .054 | .025 | .479 |
| Parental education |  |  |  |
| Low | -.369 | -.037 | .297 |
| Medium | .138 | .017 | .629 |
| High | .220 | .024 | .503 |
| Migration background | .467 | .040 | .260 |
| Depressive symptoms at baseline | .655 | .595 | <.001 |
| Anxiety at baseline | .419 | .452 | <.001 |
| **Risk factors** |  |  |  |
| Family conflicts | 1.810 | .197 | <.001 |
| School burden | 1.049 | .124 | <.001 |
| Parental depressive symptoms | .325 | .390 | <.001 |
| **Resource factors** |  |  |  |
| Personal resources | -.601 | -.405 | <.001 |
| Family climate | -.557 | -.320 | <.001 |
| Social support | -.468 | -.326 | <.001 |

*Note.* Outcome: depressive symptoms; *n* = 811.

Supplementary Table 2. Results of the univariate linear regression analyses; Risk and resource factors in children and adolescents predicting anxiety six months later

|  |  | | |
| --- | --- | --- | --- |
|  | ***b*** | **ß** | ***p*** |
| **Control variables** |  |  |  |
| Gender (female) | 1.429 | .162 | <.001 |
| Age | -.018 | -.008 | .829 |
| Parental education |  |  |  |
| Low | -.357 | -.032 | .356 |
| Medium | .232 | .026 | .456 |
| High | -.020 | -.002 | .956 |
| Migration background | .216 | .017 | .633 |
| Depressive symptoms at baseline | .578 | .481 | <.001 |
| Anxiety at baseline | .651 | .644 | <.001 |
| **Risk factors** |  |  |  |
| Family conflicts | 1.410 | .141 | <.001 |
| School burden | .871 | .094 | .007 |
| Parental depressive symptoms | .242 | .265 | <.001 |
| **Resource factors** |  |  |  |
| Personal resources | -.628 | -.388 | <.001 |
| Family climate | -.524 | -.276 | <.001 |
| Social support | -.373 | -.238 | <.001 |

*Note.* Outcome: anxiety; *n* = 811.
